# Nicotinic acid suppresses sebaceous lipogenesis of human sebocytes via activating hydroxycarboxylic acid receptor 2 (HCA_2_)

**DOI:** 10.1111/jcmm.14505

**Published:** 2019-07-05

**Authors:** Arnold Markovics, Kinga Fanni Tóth, Katalin Eszter Sós, József Magi, Adrienn Gyöngyösi, Zoltán Benyó, Christos C. Zouboulis, Tamás Bíró, Attila Oláh

**Affiliations:** ^1^ Department of Physiology, Faculty of Medicine University of Debrecen Debrecen Hungary; ^2^ Laboratory of Cerebral Cortex Research, Institute of Experimental Medicine Hungarian Academy of Sciences Budapest Hungary; ^3^ Department of Immunology, Faculty of Medicine University of Debrecen Debrecen Hungary; ^4^ Institute of Clinical Experimental Research Semmelweis University Budapest Hungary; ^5^ Departments of Dermatology, Venereology, Allergology and Immunology, Dessau Medical Center Brandenburg Medical School Theodor Fontane Dessau Germany; ^6^ DE‐MTA “Lendület” Cellular Physiology Research Group, Department of Immunology, Faculty of Medicine University of Debrecen Debrecen Hungary; ^7^ HCEMM Ltd. Szeged Hungary

**Keywords:** acne, hydroxycarboxylic acid receptor 2, nicotinic acid, sebaceous lipogenesis, sebocyte, seborrhea

## Abstract

Nicotinic acid (NA) activates hydroxycarboxylic acid receptor 2 (HCA_2_), and it is widely used in treating dyslipidaemias. Since its side effects include skin dryness, whereas its deficiency can be accompanied by dyssebacia, characterized by sebaceous gland enlargement, we asked if HCA_2_ is expressed on human sebocytes, and if NA influences sebocyte functions. By using human immortalized SZ95 sebocytes, we found that non‐cytotoxic (≤100 μmol/L; MTT‐assay) concentrations of NA had no effect on the homeostatic sebaceous lipogenesis (SLG; Nile Red), but normalized excessive, acne‐mimicking SLG induced by several lipogenic agents (arachidonic acid, anandamide, linoleic acid + testosterone; Nile Red; 48‐hr treatments). Moreover, it exerted significant anti‐proliferative actions (CyQUANT‐assay), and increased [Ca^2+^]_IC_ (Fluo‐4 AM‐based Ca^2+^‐measurement). Although NA did not prevent the lipopolysaccharide‐induced pro‐inflammatory response (up‐regulation [Q‐PCR] and release [ELISA] of several pro‐inflammatory cytokines) of the sebocytes, collectively, these data support the concept that NA may be effective in suppressing sebum production in vivo. While exploring the mechanism of the sebostatic actions, we found that sebocytes express HCA_2_ (Q‐PCR, immunofluorescent labelling), siRNA‐mediated silencing of which prevented the NA‐induced Ca^2+^‐signal and the lipostatic action. Collectively, our data introduce NA, and HCA_2_ activators in general, as novel, potent and most likely safe sebostatic agents, with possible anti‐acne potential.

## 1 INTRODUCTION

Acne is one of the most common human skin diseases. It impairs quality of life of millions of patients world‐wide, and is characterized by excessively increased and qualitatively altered sebaceous lipogenesis (SLG), complex inflammatory processes, as well as abnormal skin—microbiota cross‐talk.^1^, ^2^, ^3^, ^4^ Although highly efficient anti‐acne agents (eg isotretinoin) exist, their administration is often limited by different (sometimes quite serious) side effects.^1^, ^2^ Thus, there is an unmet need from both the scientific community and society to develop novel, highly efficient therapeutic agents exhibiting better side effect and safety profiles.

Nicotinic acid (NA) and nicotinamide are members of the vitamin B3 complex, whose deficiency leads to pellagra, a disease classically characterized by diarrhoea, dementia, dermatitis, and, if remains untreated, death. Interestingly, in light‐exposed skin areas with high levels of sebum excretion, such as the face, dyssebacia (a sebaceous gland [SG] dysfunction characterized by abnormal, inspissated sebum production, as well as consequently enlarged facial SGs and dilated SG orifices through which the plugs may be projected) has also been reported.^5^, ^6^, ^7^ Importantly, in pharmacological doses, NA has widely been used in the clinical practice in treating dyslipidaemias for decades, while, quite surprisingly, nicotinamide (albeit being equipotent with NA as a vitamin) fails to improve these conditions.^8^, ^9^ Interestingly, clinical administration of NA is limited by a harmless, but quite inconvenient and frequently occurring side effect, namely the NA‐induced skin flush response, which consists of cutaneous vasodilation accompanied by itch and a burning sensation mainly affecting the face and the upper body. Importantly, we have previously demonstrated that the flush is mediated via the NA‐induced activation of hydroxycarboxylic acid receptor 2 (HCA_2_; previously known as “HM74A”, “niacin receptor 1”, “GPR109A” or in mice as “PUMA‐G”) expressed by epidermal Langerhans cells.^10^, ^11^

HCA_2_ is a G_i_‐coupled receptor abundantly expressed in adipocytes and in various immune cells, where it is involved in orchestrating anti‐inflammatory actions.^12^, ^13^, ^14^, ^15^ Interestingly, in a striking contrast with NA, nicotinamide has only negligible affinity to HCA_2_, which explains its aforementioned inefficiency in the clinical management of dyslipidaemias.^9^, ^16^, ^17^

Importantly, when applied at pharmacological doses, side effects of NA include skin dryness (https://www.drugs.com/pro/niacin.html), whereas NA deficiency can be accompanied by sebaceous gland enlargement.^6^ Moreover, pilot clinical studies demonstrate that high doses of orally administered NA may exert beneficial effects against acne.^18^, ^19^, ^20^ Thus, based on the aforementioned data, we hypothesized that NA may directly impact on the biology of human sebocytes, and that these actions might be mediated via HCA_2_ receptors.

In order to challenge this working hypothesis, we tested effects of NA, and putative expression of HCA_2_. Specifically, we asked if NA influences (a) basal (homeostatic) SLG; (b) acne mimicking, excessive SLG; (c) proliferation; and (d) immune behaviour of cultured human sebocytes. Moreover, we aimed to assess expression of HCA_2_ and its putative role in mediating the above actions. Considering the regrettable limitations of the available model systems to study sebaceous gland biology, including ineffective culturing of primary human sebocytes and the lack of adequate animal model systems,^3^, ^21^, ^22^ we decided to use the human immortalized SZ95 sebocyte cell line,^22^, ^23^ a widely accepted and reliable model to study human sebaceous gland biology in vitro.^2^, ^3^, ^22^, ^23^

## 2 MATERIALS AND METHODS

### 2.1 Materials

Anandamide (AEA) and arachidonic acid (AA) were purchased from Cayman Chemical Company (Ann Arbor, MI, USA); γ‐irradiated lipopolysaccharide from *Escherichia coli* 026:B6 (LPS), linoleic acid (LA), ruthenium red (RR), testosterone (T) and nicotinic acid (NA) were obtained from Sigma‐Aldrich (St. Louis, MO, USA), whereas BAPTA AM was acquired from Tocris (Bio‐Techne; Minneapolis, MN, USA). AEA, AA and LA were dissolved in absolute ethanol, whereas the solvent for BAPTA AM, T and RR was dimethyl sulphoxide (DMSO). LPS was dissolved in filtered distilled water, whereas NA was diluted directly in the culture medium.

### 2.2 Cell culturing

Human immortalized SZ95 sebocytes, originated from human facial sebaceous glands,^23^ were cultured as described previously.^24^, ^25^ Briefly, Sebomed^®^ Basal Medium (Biochrom, Berlin, Germany) was supplemented with 10 (V/V)% fetal bovine serum (Life Technologies Hungary Ltd, Budapest, Hungary), 1 mmol/L CaCl_2_, 5 ng/ml human epidermal growth factor (Sigma‐Aldrich) and MycoZap™ Plus‐CL (1:500; Lonza, Budapest, Hungary). The medium was changed every other day, and cells were subcultured at 60%‐70% confluence.

### 2.3 Determination of intracellular lipids

For quantitative measurement of sebaceous (neutral) lipid content, cells (20 000 cells/well) were cultured in 96‐well “black well/clear bottom” plates (Greiner Bio‐One, Frickenhausen, Germany) in quadruplicates, and were treated with compounds as indicated.^24^, ^25^, ^26^ Subsequently, supernatants were discarded, cells were washed twice with phosphate‐buffered saline (PBS; 115 mmol/L NaCl, 20 mmol/L Na_2_HPO_4_, pH 7.4; all from Sigma‐Aldrich), and 100 µl of a 1 µg/ml Nile Red (Sigma‐Aldrich) solution in PBS was added to each well. The plates were then incubated at 37°C for 30 minutes, and fluorescence was measured on FlexStation 3 multimode microplate reader (Molecular Devices, San Francisco, CA, USA). Results, measured in relative fluorescence units, are expressed as percentage of the vehicle control regarded as 100%, using 485 nm excitation and 565 nm emission wavelengths.

### 2.4 Determination of cellular viability

The viability of the cells was determined by MTT‐assay (Sigma‐Aldrich) measuring the conversion of the tetrazolium salt to formazan by mitochondrial dehydrogenases.^24^, ^25^, ^26^, ^27^, ^28^ Cells were plated in 96‐well plates (20 000 cells/well) in quadruplicates, and were treated as indicated. Cells were then incubated with 0.5 mg/ml MTT reagent for 3 hours, and concentration of formazan crystals (as an indicator of number of viable cells) was determined colorimetrically at 565 nm by using FlexStation 3 multi‐mode microplate reader (Molecular Devices). Results were expressed as percentage of vehicle control regarded as 100%.

### 2.5 Determination of apoptosis

A decrease in the mitochondrial membrane potential is one of the earliest markers of apoptosis. Therefore, to assess the process, mitochondrial membrane potential of SZ95 sebocytes was determined using a MitoProbe™ DilC_1_(5) Assay Kit (Life Technologies Hungary Ltd.). 24, 25, 26 Cells (20 000 cells/well) were cultured in 96‐well “black well/clear bottom” plates (Greiner Bio One) in quadruplicates and were treated as indicated. After removal of supernatants, cells were incubated for 30 minutes with DilC_1_(5) working solution (50 μl/well), then washed with PBS, and the fluorescence of DilC_1_(5) was measured at 630 nm excitation and 670 nm emission wavelengths using FlexStation 3 multi‐mode microplate reader (Molecular Devices). Relative fluorescence values were expressed as percentage of vehicle control regarded as 100%. As a positive control for apoptosis, we applied carbonyl cyanide m‐chlorophenyl hydrazone (CCCP; Life Technologies Hungary Ltd.) dissolved in the DilC_1_(5) working solution (1:200 for 30 minutes at 37°C).

### 2.6 Determination of necrosis

Necrotic processes were determined by SYTOX Green staining (Life Technologies Hungary Ltd.).^24^, ^25^, ^26^ The dye is able to penetrate (and then to bind to the nucleic acids) only to necrotic cells with ruptured plasma membranes, whereas healthy cells with intact surface membranes show negligible SYTOX Green staining intensity. Cells were cultured in 96‐well “black well/clear bottom” plates (Greiner Bio One), and treated as indicated. Supernatants were then discarded, and the cells were incubated for 30 minutes with 1 μmol/L SYTOX Green dye. Following incubation, cells were washed with PBS, the culture medium was replaced, and fluorescence of SYTOX Green was measured at 490 nm excitation and 520 nm emission wavelengths using FlexStation 3 multi‐mode microplate reader (Molecular Devices). As a positive control for necrosis, lysis buffer (LB; 1:100 in the SYTOX Green working solution for 30 minutes at 37°C; Life Technologies Hungary Ltd.) was applied. Relative fluorescence values were expressed as percentage of positive control regarded as 100%. Due to their spectral properties, DilC_1_(5) and SYTOX Green dyes were always administered together, enabling us to investigate necrotic and early apoptotic processes of the same cultures. Selective decrease of DilC_1_(5) intensity indicated mitochondrial depolarization (ie the onset of early apoptotic processes), whereas increase of SYTOX Green staining intensity revealed necrotic cell death.

### 2.7 Determination of cellular proliferation

The degree of cellular growth (reflecting proliferation) was determined by measuring the DNA content of cells using CyQUANT Cell Proliferation Assay Kit (Life Technologies Hungary Ltd).^24^, ^26^, ^27^ SZ95 sebocytes (2000 cells/well) were cultured in 96‐well “black well/clear bottom” plates (Greiner Bio‐One) and were treated as indicated. Supernatants were then removed by blotting on paper towels, and the plates were subsequently frozen at −80°C. The plates were then thawed at room temperature, and 200 µl of CyQUANT dye/cell lysis buffer mixture was added to each well. After 5 minutes of incubation, fluorescence was measured at 490 nm excitation and 520 nm emission wavelengths using FlexStation 3 multimode microplate reader (Molecular Devices). Relative fluorescence values were expressed as percentage of 24‐hr vehicle control regarded as 100%.

### 2.8 RNA isolation, reverse transcription and quantitative ‘real‐time’ PCR (Q‐PCR)

Q‐PCR was performed on a Roche LightCycler 480 System (Roche, Basel, Switzerland) using the 5′ nuclease assay.^25^, ^29^ Total RNA was isolated using TRIzol (Life Technologies Hungary Ltd.), DNase treatment was performed according to the manufacturer's protocol, and then, 1 µg of total RNA was reverse‐transcribed into cDNA using High‐Capacity cDNA Kit from Life Technologies Hungary Ltd. PCR amplification was performed using the TaqMan^®^ Gene Expression Assays (assay IDs: Hs00174092_m1 for *interleukin [IL]‐1α*, Hs00174097_m1 for *IL‐1β*, Hs00985639_m1 for *IL‐6*, Hs00174103_m1 for *IL‐8*, Hs00174128_m1 for *tumour necrosis factor [TNF]‐α*; Hs00271958_s1 for *H* *C* *A*_*2*_) and the TaqMan universal PCR master mix protocol (Applied Biosystems). As internal controls, transcripts of *18S RNA* (assay ID: Hs03928985_g1) or *glyceraldehyde 3‐phosphate dehydrogenase* (*GAPDH*; assay ID: Hs99999901_s1) were determined. The amount of the transcripts was normalized to those of the housekeeping gene using the ΔCT method. Finally, when indicated, the relative expression values were further normalized to the ones of the vehicle‐treated or scrambled RNA‐transfected controls (ΔΔCT method).

### 2.9 Determination of cytokine release (ELISA)

Cells were seeded in 35 mm culture dishes (500 000 cells/1.5 ml medium/ dish), and were treated as indicated for 3 or 24 hours.^24^, ^25^, ^29^ Supernatants were collected, and the released amount of IL‐6 and IL‐8 cytokines was determined using OptEIA kits (BD Pharmingen, Franklin Lakes, NJ, USA) according to the manufacturer's protocol.

### 2.10 Fluorescent Ca^2+^ measurements

Human SZ95 sebocytes were seeded in 96‐well “black well/clear bottom” plates (Greiner Bio‐One) at a density of 20 000 cells/well resulting in an almost confluent culture, and treatments were initiated in the subsequent day.^26^ The cells were washed once with 1% bovine serum albumin and 2.5 mmol/L probenecid (both from Sigma‐Aldrich) containing Hank's solution (136.8 mmol/L NaCl, 5.4 mmol/L KCl, 0.34 mmol/L Na_2_HPO_4_, 0.44 mmol/L KH_2_PO_4_, 0.81 mmol/L MgSO_4_, 1.26 mmol/L CaCl_2_, 5.56 mmol/L glucose, 4.17 mM NaHCO_3_, pH 7.2, all from Sigma‐Aldrich), and then were loaded with 1 μmol/L Fluo‐4 AM (Life Technologies Hungary Ltd.) dissolved in Hank's solution (100 μl/well) at 37°C for 30 minutes. The cells were then washed three times with Hank's solution (100 μl/well). In the case of the measurements performed in “Low [Ca^2+^]_EC_”, CaCl_2_ was omitted from the buffer, and was substituted by equimolar glucose. The plates were then placed into a FlexStation 3 multi‐mode microplate reader (Molecular Devices), and alterations of the cytoplasmic Ca^2+^ concentration (reflected by changes in fluorescence; λ_EX_: 490 nm, λ_EM_: 520 nm) were monitored following the application of compounds in the indicated concentrations. In order to probe reactivity and viability of the cells, at the end of each measurement, 0.2 mg/ml ATP was administered as a positive control (data not shown). Data are presented as F/F_0_, where F_0_ is the average baseline fluorescence (ie before compound application), whereas F is the actual fluorescence.

### 2.11 siRNA transfection‐mediated selective gene silencing

Human SZ95 cells were seeded in (d = 35 mm) Petri dishes or in 96‐well “black well/clear bottom” plates (Greiner Bio‐One) or on glass coverslips in 6‐well plates in culture medium. On the other day, medium was changed, and the cells were transfected with siRNA oligonucleotides targeting human HCA_2_ (Stealth RNAi, assay ID: HSS155269; Life Technologies Hungary Ltd.) using Lipofectamine^®^ RNA_i_ MAX Transfection Reagent and serum‐free Opti‐MEM™ (both from Life Technologies Hungary Ltd.). For controls, siRNA Negative Control Duplexes with medium GC ratio (SCR, Life Technologies Hungary Ltd.) were employed. Silencing efficiency was monitored on post‐transfection Days 2 and 3 at the mRNA (Q‐PCR) and protein (immunofluorescent labelling) levels.

### 2.12 Immunofluorescent labelling

Human SZ95 sebocytes were cultured on glass coverslips in 6‐well plates. Cells were fixed by 4% paraformaldehyde containing PBS for 5 minutes at room temperature. Following appropriate washing in PBS, coverslips were incubated for 30 minutes in blocking solution (3 [V/V]% bovine serum albumin containing PBS; both from Sigma‐Aldrich) at room temperature. Following appropriate washing in PBS, cells were probed with primary antibodies raised against human HCA_2_ (dissolved in 1:50 in 1.5 [V/V]% BSA containing PBS, Santa Cruz Biotechnology, Inc, Dallas, TX, USA) for 60 minutes at room temperature. Following appropriate washing in PBS, coverslips were incubated with Alexa‐Fluor^®^‐488‐conjugated anti‐rabbit IgG Fc segment‐specific secondary antibodies (1:1000 in 1.5 [V/V]% BSA containing PBS; Cell Signaling Technology, Leiden, The Netherlands) and DAPI (1:1000, Vector Laboratories, Burlingame, CA, USA) diluted together in 1.5 (V/V)% BSA containing PBS for 1 hour at room temperature, which was followed by appropriate washing in PBS. As negative controls, the primary antibodies were omitted from the procedure. Visualization of the proteins was performed by using an Olympus IX81 fluorescent microscope (Olympus, Sindzsuku, Tokyo, Japan). When indicated, images were then subjected to subsequent image analysis to assess semi‐quantitative alterations in HCA_2_ expression. In these cases, images (taken from simultaneously performed stainings) were analysed by *Fiji* software,^30^ and mean grey values (detected in the green channel by using strictly the same settings during image exposure in all cases) were compared to each other following the subtraction of the background (ie mean grey value of the cell‐free area).

### 2.13 Statistical analysis

Data were analysed by *IBM SPSS Statics software version 20* (IBM Armonk, North Castle, NY, USA), using Student's two‐tailed, two‐sample *t* test (paired comparisons) or one‐way ANOVA with Bonferroni's *post hoc* test (multiple comparisons), and *P* < 0.05 values were regarded as significant differences. Graphs were plotted using *Origin Pro Plus 6* software (Microcal, Northampton, MA, USA).

### 2.14 Ethical approval

Data presented in the current manuscript were generated by using a human immortalized cell line. Since primary human material or animals were not used, ethical approval was not required for the study.

## 3 RESULTS

### 3.1 Up to 100 μmol/L, NA does not decrease the viability of human sebocytes

First, by using MTT‐assay, we found that up to 100 µmol/L NA did not decrease the viability of sebocytes (24‐72 hours; Figure S1). In order to exclude the possibility of the onset of early apoptotic and necrotic processes resulting in no obvious alterations in the MTT‐assay, we further assessed the effects of NA by combined fluorescent labelling (DilC_1_(5) & SYTOX Green dyes), a routinely used method in our recent sebocyte‐oriented studies.^24^, ^25^, ^26^, ^31^ Importantly, we found that in line with our MTT data, NA did not induce apoptotic or necrotic events (≤100 μmol/L; 24‐ and 48‐hr treatments; Figure S2), confirming that in this time window and concentration range, it can indeed be administered without the risk of any biologically relevant cytotoxic actions.

### 3.2 NA does not influence homeostatic SLG, but normalizes various lipogenic agent‐induced, excessive, acne‐mimicking lipid synthesis

**Figure 1:**
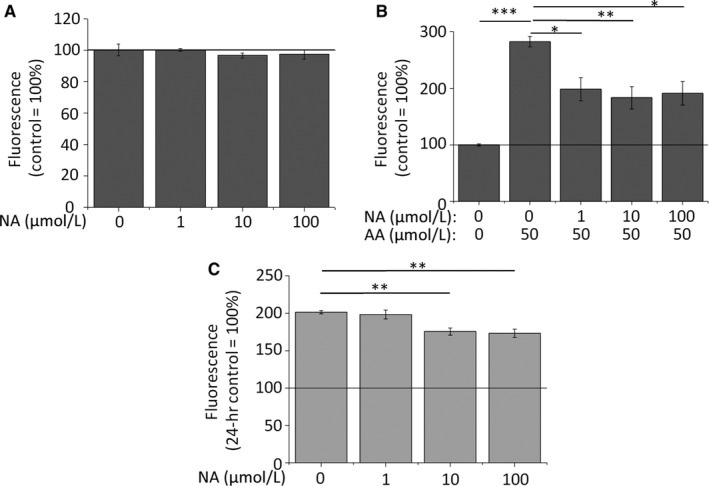
NA does not influence homeostatic SLG, but normalizes AA‐induced, excessive, acne‐mimicking lipid production. (A‐B) Nile Red assay. SLG of SZ95 sebocytes was assessed following 48‐hr treatments. Results are expressed in the percentage of the vehicle control (100%, solid line) as mean ± SEM of four independent determinations. Two additional experiments yielded similar results. *, ** and *** mark significant (*P* < 0.05, 0.01 and 0.001, respectively) differences, as indicated. (C) CyQUANT‐assay. Sebocytes were plated at low (2000/well) initial cell count to enable rapid proliferation. Cell count was assessed following 72‐hr treatments. Results are expressed in the percentage of the 24‐hr vehicle control (100%, solid line) as mean ± SEM of four independent determinations. Two additional experiments yielded similar results. ** marks significant (*P* < 0.01) differences, as indicated. AA, arachidonic acid; NA, nicotinic acid

Next, by using fluorescent Nile Red staining, we aimed to study the effects of NA on the most characteristic biological function of the sebocytes, ie the basal, homeostatic SLG. We found that, when administered at the above‐determined non‐cytotoxic concentrations, NA did not influence basal SLG in course of 48‐hr treatments (Figure 1A).

Since one of the most obvious steps of the pathogenesis of acne is seborrhea,^1^, ^2^, ^3^ next we asked if NA influences pro‐acne agent‐induced, excessive, acne‐mimicking SLG. To test this, we administered arachidonic acid (AA), a pro‐inflammatory lipid mediator involved in the pathogenesis of acne,^1^, ^2^, ^3^, ^4^ which promotes SLG mostly via activating protein kinase C δ.^32^ As expected, AA (50 μmol/L) was able to induce a marked elevation in the SLG over the course of 48‐hr treatments. This lipogenic effect was significantly suppressed by all the above‐determined non‐cytotoxic concentrations of NA with similar efficiency (Figure 1B), indicating that NA may be efficient in normalizing excessive SLG in acne.

To assess if this lipogenesis‐reducing activity is specific for AA, or if it rather reflects activation of a universal lipostatic signalling pathway, we probed NA’s efficiency against such stimuli, which are known to activate partially independent lipogenic signalling pathways. To this end, the endocannabinoid anandamide (AEA; 30 μmol/L),^33^, ^34^, ^35^ an endogenous lipid mediator, which was shown to promote SLG via activating CB_2_ cannabinoid receptor,^31^ and the combination of linoleic acid and testosterone (LA + T in 100 and 1 μmol/L, respectively) targeting peroxisome proliferator‐activated receptors (PPARs)^36^ were applied. Of great importance, NA suppressed both lipogenic stimuli, irrespective of the underlying signalling pathways (Figure S3A‐B), supporting the concept that NA is a universally effective lipostatic agent (ie it can normalize excessive SLG irrespective of the actual lipogenic pathway), which might even be of therapeutic value in acne.

### 3.3 NA exerts anti‐proliferative actions, but does not influence lipopolysaccharide (LPS)‐induced pro‐inflammatory response of the sebocytes

Considering that sebum production is realized via holocrine secretion, its clinical level does not only depend on the lipid production of the individual sebocytes, but also on the number of the lipid producing cells.^2^, ^3^, ^37^, ^38^, ^39^ Thus, a potent anti‐acne agent is also expected to suppress proliferation of sebocytes. In order to enable rapid proliferation, SZ95 sebocytes were plated at low initial density (2000 cells/well), and treated by NA (1‐100 μmol/L) for 72 hours. Importantly, NA exerted a significant and concentration‐dependent anti‐proliferative effect, ie it suppressed cell growth compared to the daily vehicle control group, but unlike the lipostatic effects, anti‐proliferative actions only developed at higher (10‐100 μmol/L) concentrations (Figure 1C). It is also noteworthy that perfectly in line with the viability data (Figure S1‐S2), the cell count did not decrease below the level of the 24‐hr control, arguing for the onset of a pure anti‐proliferative action without any detrimental effects on the viability.

**Figure 2:**
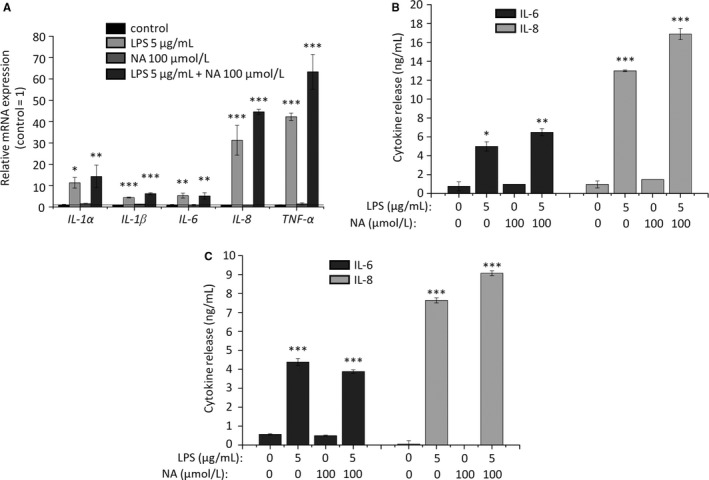
NA does not reduce cytokine expression and release of human sebocytes. (A) Q‐PCR *IL‐1α*, *IL‐1β*, *IL‐6*, *IL‐8* and *TNF‐α* mRNA expressions were determined following 3‐hr LPS‐treatment with or without NA. Data are presented by using ΔΔCT method regarding *18S RNA*‐normalized mRNA expressions of the vehicle control as 1 (solid line). Data are expressed as mean ± SEM of 2‐3 determinations. One additional experiment yielded similar results. ** and ***P < 0.01 and 0.001, respectively, as indicated. (B, C) ELISA. IL‐6 and IL‐8 content of the sebocyte supernatants was determined following 3‐hr (B) and 24‐hr (C) LPS‐treatment with or without NA. Data are expressed as mean ± SEM of three determinations. One additional experiment yielded similar results. *, **, and *** mark significant (*P* < 0.05, 0.01 or 0.001, respectively) differences compared to the vehicle control. LPS, lipopolysaccharide; NA, nicotinic acid

Besides the pathologically elevated SLG, another important contributor to the development of acne is inflammation^1^, ^2^, ^3^, ^4^; thus, we investigated the effects of NA on the immune behaviour of the sebocytes. Pro‐inflammatory response was evoked by 5 μg/ml lipopolysaccharide (LPS) according to our previously optimized protocol.^24^, ^25^ As expected, 3‐hr LPS treatment was able to induce a strong up‐regulation of various pro‐inflammatory cytokines at the mRNA level, including *interleukin* (*IL*)‐*1α*, *IL‐1β*, *IL‐6*, *IL‐8* and *tumour necrosis factor‐α* (*TNF‐α*) (Q‐PCR; Figure 2A). We found that 100 μmol/L NA did not suppress the LPS‐induced up‐regulation of the above cytokines, and it had no effect when it was administered alone either (Figure 2A). In order to get a deeper insight to the effects of NA on the inflammatory responses, we also measured release of IL‐6 and IL‐8, two key cytokines, which, upon being secreted by the sebocytes, are involved in the regulation of Th_17_ polarization.^40^ In line with the mRNA level data, ELISA measurement of the supernatants following 3‐hr (to assess early release of the preformed cytokine pool) and 24‐hr treatments (to observe release of de novo synthesized cytokines) did not reveal any NA‐mediated effects on the cytokine release (Figure 2B‐C). Thus, our data support the concept that NA is unlikely to directly influence immune behaviour of human sebocytes.

### 3.4 Lipostatic effects of NA are mediated via the activation of a Ca^2+^‐dependent signalling pathway

**Figure 3:**
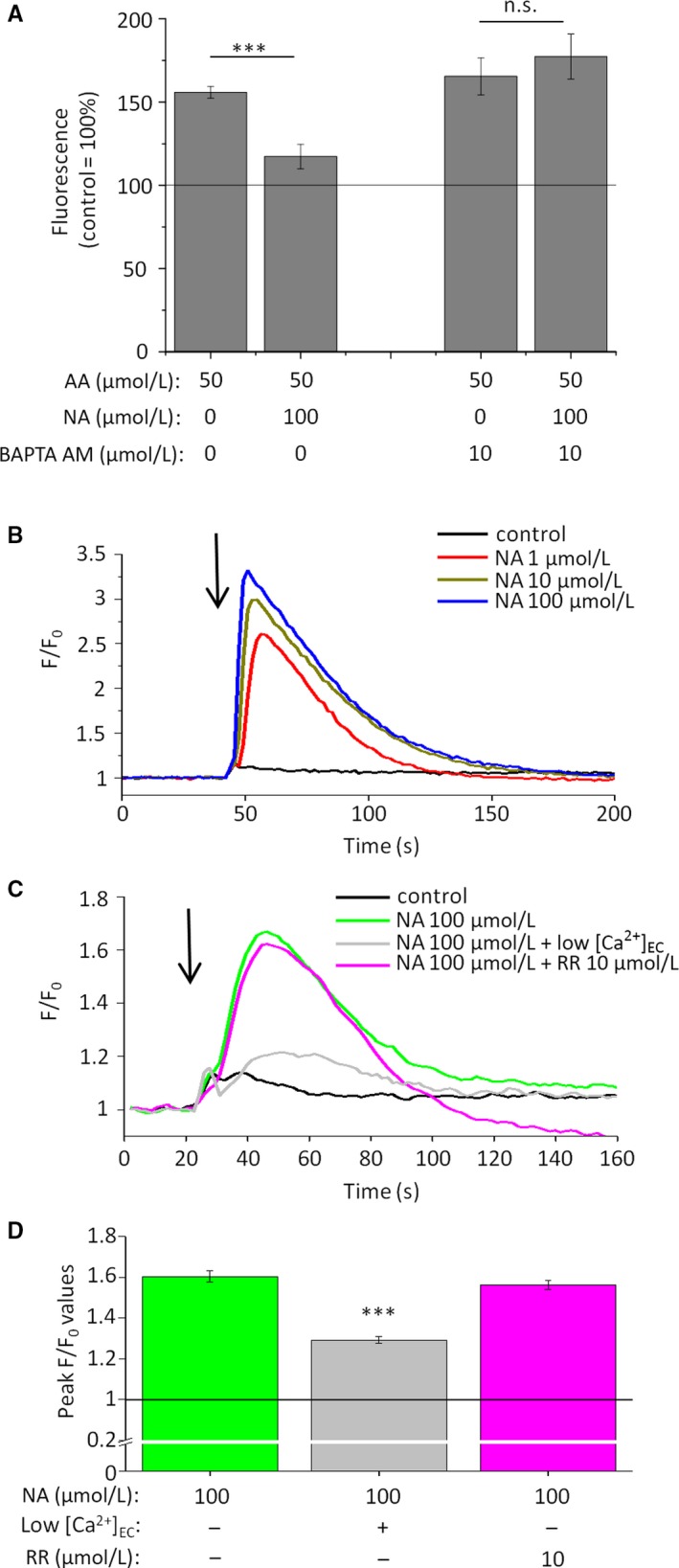
Lipostatic effects of NA are Ca^2+^‐dependent ones, and NA increases [Ca^2+^]_IC_ of human sebocytes in a concentration‐dependent manner, via activating surface membrane non‐TPRV Ca^2+^‐channels. (A) Nile Red assay. SLG of SZ95 sebocytes was assessed following 48‐hr treatments. Results are expressed in the percentage of the vehicle control (100%, solid line) as mean ± SEM of four independent determinations. One additional experiments yielded similar results. *** marks significant (*P* < 0.001) difference, as indicated. n.s.: not significant. (B‐C) Fluo‐4 AM‐based fluorescent Ca^2+^‐measurement. Compounds were applied as indicated by the arrows. Fluorescence (measured in relative fluorescence units) was normalized to the baseline. “Low [Ca^2+^]_EC_” indicates the use of nominally Ca^2+^‐free Hank's solution. One additional experiment yielded similar results. (D) Statistical analysis of the peak fluorescence (F/F_0_) values shown on panel (C). Data are presented as mean ± SEM of N = 12 independent determinations. ***P < 0.001 compared to the NA treatment in normal [Ca^2+^]_EC_. NA: nicotinic acid; RR: ruthenium red (pan‐TRPV channel blocker)

Next, we intended to unveil the mechanism of the lipid synthesis reducing activity. Since elevation of the [Ca^2+^]_IC_ often leads to lipostatic actions similar to the ones described above,^26^, ^41^, ^42^, ^43^ and it is well‐documented that administration of NA elevates [Ca^2+^]_IC_ in certain cell types,^10^, ^11^ we first asked if alterations of [Ca^2+^]_IC_ play any role in mediating lipostatic effects of NA. Importantly, we found that lipostatic effects could be prevented by the co‐administration of the cell‐permeable Ca^2+^‐chelator BAPTA AM (Figure 3A), indicating that, similar to eg CBD, NA also suppressed SLG via activating a Ca^2+^‐coupled signalling pathway.

### 3.5 Lipostatic concentrations of NA increase [Ca^2±^]_IC_ via activating surface membrane non‐TRPV Ca^2±^ channels

Next, we found that potent lipostatic and anti‐proliferative concentrations of NA were able to substantially and concentration‐dependently increase [Ca^2+^]_IC_ (Fluo‐4 AM‐based fluorescent Ca^2+^‐measurement; Figure 3B), most likely via activating plasma membrane calcium channels, since the signal was almost completely absent in nominally calcium‐free medium (Figure 3C‐D). Considering that NA was shown to directly activate certain calcium‐permeable transient receptor potential ion channels belonging to the vanilloid (TRPV) subclass,^44^, ^45^, ^46^ and that certain TRPV channels were demonstrated to suppress SLG,^26^, ^42^, ^43^ we probed their involvement in the onset of the NA‐induced calcium signal by using ruthenium red (RR), a well‐known pan‐antagonist of the TRPV channels.^47^ Importantly, we found that RR was unable to modify the NA‐induced calcium signals (Figure 3B‐C) indicating that they are likely to be mediated via yet unknown, non‐TRPV surface membrane calcium permeable pores.

### 3.6 Sebocytes express HCA_2_ receptor, which selectively mediates lipostatic and [Ca^2±^]_IC_‐increasing effects of NA

**Figure 4:**
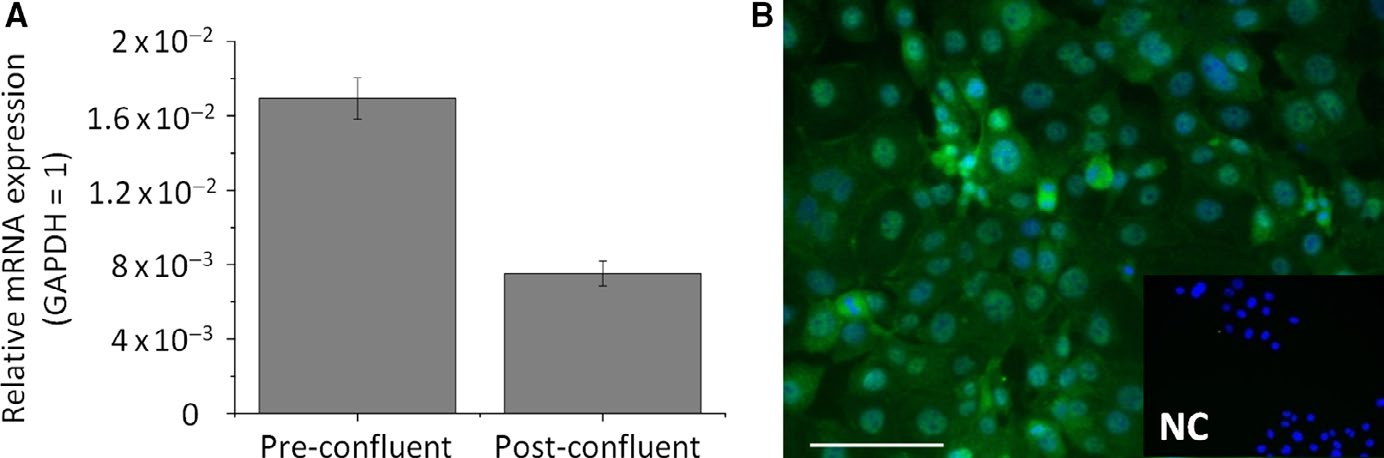
HCA_2_ receptor is expressed in cultured human sebocytes. (A) Q‐PCR. Sebocytes were harvested at different confluences. *H* *C* *A*_*2*_ expression was normalized to the level of *glyceraldehyde‐3‐phosphate dehydrogenase* (*GAPDH*) of the same sample, and are expressed as mean ± SD of three determinations. (B) Immunofluorescent labelling. HCA_2_ immunoreactivity was determined by immunofluorescence labelling (Alexa‐Fluor^®^‐488, green) in SZ95 sebocytes. Nuclei were counterstained by DAPI (blue). NC: negative control. Scale bar: 20 μm

**Figure 5:**
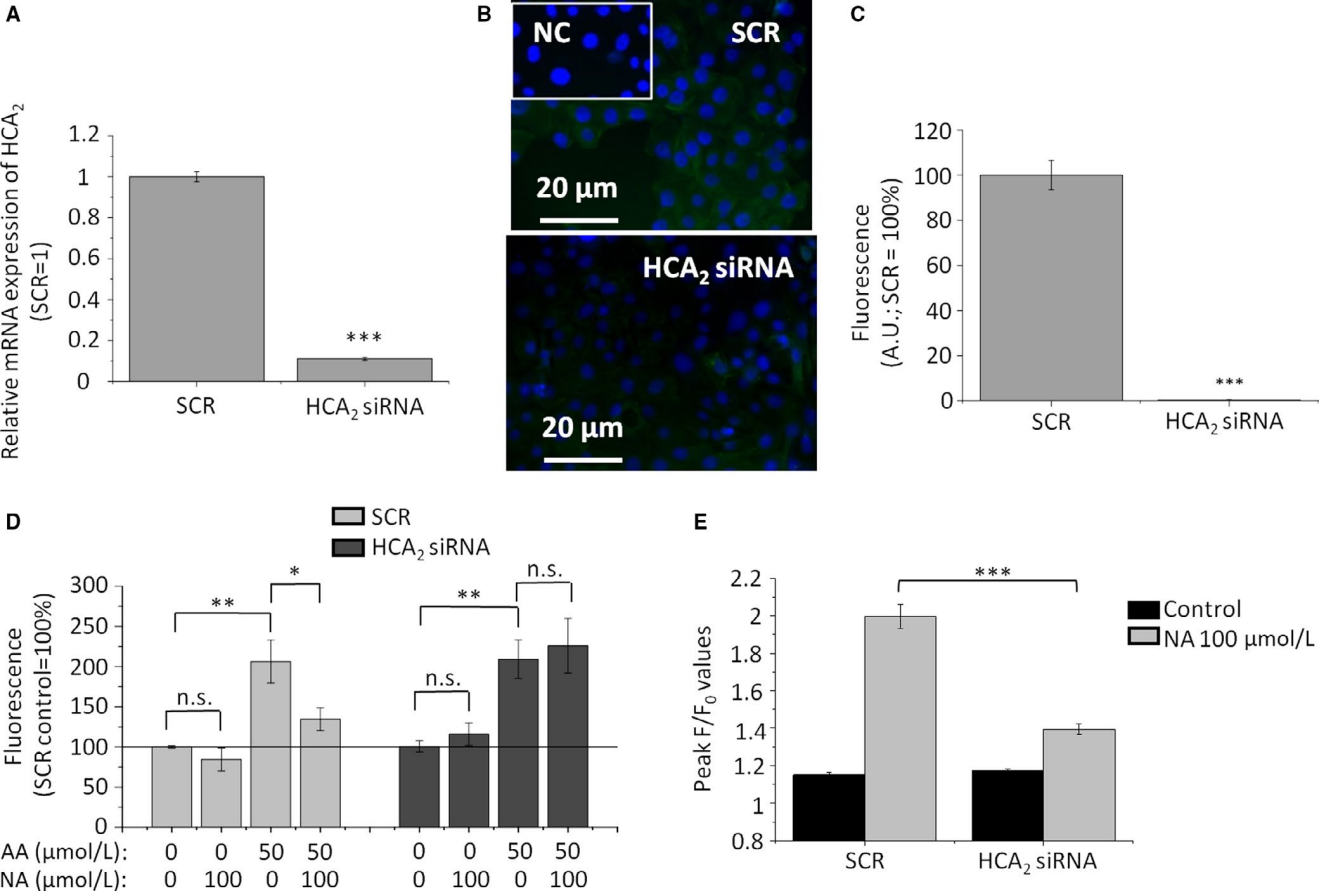
Silencing of HCA_2_ prevents lipostatic and [Ca^2+^]_IC_‐elevating effects of NA. (A) Q‐PCR. Sebocytes were harvested on post‐transfection Day 2. HCA_2_ expression was first normalized to the level of *18S RNA* of the same sample, and then relative mRNA expression was normalized to the one of the SCR control group. Data are expressed as mean ± SD of three determinations. (B) Immunofluorescent labelling. HCA_2_ expression (green) was assessed on post‐transfection Day 3. Nuclei were counterstained with DAPI (blue). Scale bars: 20 μm. (C) Image analysis of the immunofluorescent labelling on post‐transfection Day 3. Following appropriate background subtraction (for details, see the Materials and methods section) data of the green channel were expressed in the percentage of the SCR control, and presented as mean ± SEM of N = 93 cells in altogether 2‐4 visual fields from at least two coverslips in each group. ***P < 0.001. AU: arbitrary units. SCR: non‐sense (scrambled) RNA construct‐transfected cells. (D) SLG assessed by Nile Red assay on SCR‐transfected as well as HCA_2_‐silenced sebocytes. 48‐hr treatment of the sebocytes was initiated on post‐transfection Day 2. Results are expressed in the percentage of the SCR vehicle control (100%, solid line) as mean ± SEM of four independent determinations. Two additional experiments yielded similar results. (E) Statistical analysis of Fluo‐4 AM‐based fluorescent Ca^2+^‐measurement performed on post‐transfection Day 2. Fluorescence (measured in relative fluorescence units) was normalized to the baseline. The peak fluorescence (F/F_0_) values are presented as mean ± SEM of N = 7 independent determinations. One additional experiment yielded similar results. *, ** and *** mark significant (*P* < 0.05, 0.01 and 0.001, respectively) differences, as indicated. AA: arachidonic acid; HCA_2_ siRNA: HCA_2_‐silenced cells; NA: nicotinic acid; SCR: non‐sense RNA‐transfected scrambled control cells

Since the above functional data on the calcium homeostasis made the involvement of TRPV channels in mediating lipostatic actions of NA highly unlikely, we asked if the major NA receptor, ie HCA_2_, contributes to this process. Importantly, we found that HCA_2_ is indeed expressed by human sebocytes both at the mRNA (Q‐PCR; Figure 4A) and protein levels (immunofluorescence; Figure 4B). Next, in the lack of potent and selective antagonists,^14^ siRNA‐mediated selective gene silencing was applied, which resulted in a significant decrease in both mRNA (Figure 5A) and protein (Figure 5B‐C) expression of HCA_2_. Moreover, as revealed by Nile Red assays simultaneously executed on non‐sense RNA‐transfected scrambled control as well as HCA_2_‐silenced sebocytes, lipostatic action of NA was selectively mediated via activating HCA_2_, since silencing of the receptor completely abrogated it (Figure 5D). Intriguingly, HCA_2_‐silencing had no obvious impact on AA‐induced lipogenesis (Figure 5D), and neither AA, nor NA treatment influenced HCA_2_ expression of un‐transfected sebocytes (Figure S4). Last, but not least, we could also demonstrate that siRNA‐mediated silencing of HCA_2_ prevented the NA‐induced transient elevation of the [Ca^2+^]_IC_ (Figure 5E), supporting the concept that, similar to eg Langerhans cells,^10^, ^11^ it was coupled to HCA_2_‐activation in sebocytes as well.

## 4 DISCUSSION

**Figure 6:**
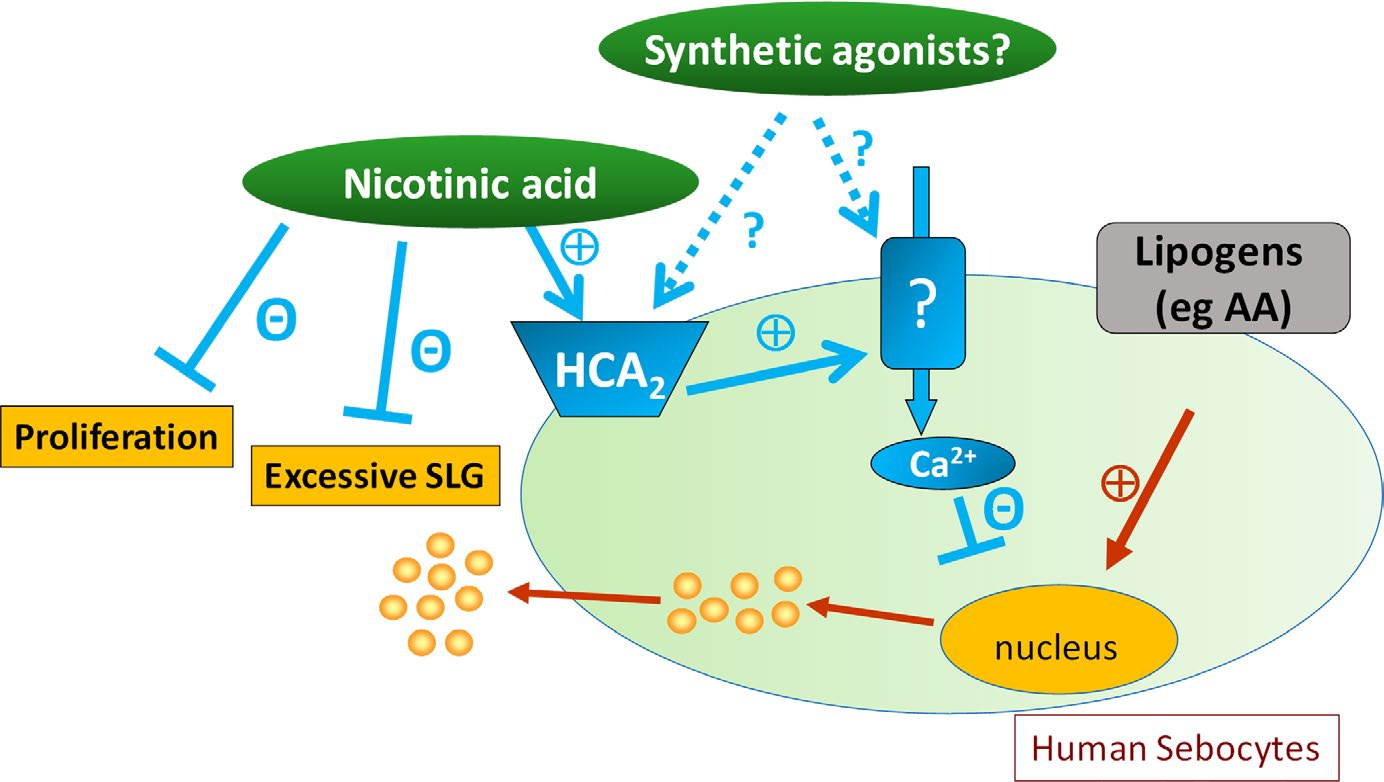
Overview of the anti‐acne potential of NA and HCA_2_‐agonism*.* Note that NA does not decrease basal, homeostatic lipid synthesis or viability of human sebocytes, but it is highly and universally efficient against lipogen‐induced, excessive, acne‐mimicking SLG, and exerts anti‐proliferative actions. The anti‐acne potential of NA, as well as of pharmacological HCA_2_ agonists and activators of the so far unidentified surface membrane calcium channels is to be probed in targeted future clinical trials

In the current study we provide evidence that, without influencing basal, homeostatic lipid synthesis (Figure 1A) or viability (Figures S1 and S2) of human sebocytes, NA normalizes acne‐mimicking, excessive SLG induced by various pro‐acne agents (Figure 1B; Figure S3), and exerts concentration‐dependent anti‐proliferative effects (Figure 1C). These actions are realized via the activation of a previously unknown regulator of the sebocyte biology, the HCA_2_ receptor (Figures 3, 4, 5). Thus, our data not only highlight the possibility of dermatological use of NA, a well‐characterized lipid‐lowering substance having a long clinical history, and exhibiting acceptable side effect and safety profiles, but also introduce a previously unknown, attractive and druggable pharmacological target (ie HCA_2_ receptor) for future sebostatic and anti‐acne drug development (Figure 6). Well‐controlled clinical trials by using appropriate topical formulations delivering NA directly to the sebaceous glands are therefore urgently invited to exploit the therapeutic potential of this sebaceous gland‐wise previously completely unexplored regulatory system.

Although to the best of our knowledge, no placebo‐controlled clinical trials were organized so far to dissect the acne‐wise relevant dermatological effects of NA, beyond our above presented data, there are several pieces of evidence further supporting the concept that it may exert beneficial effects. First, in pellagra (ie NA deficiency), dyssebacia can often be detected.^6^ Second, side effects of NA administered at pharmacological doses include skin dryness (https://www.drugs.com/pro/niacin.html). Third, results of certain pilot clinical studies suggest that orally administered NA may be beneficial against acne.^18^, ^19^, ^20^

It should also be noted that topically applied nicotinamide (the other member of the vitamin B3 complex) was found to efficiently reduce sebum production in a double‐blind, placebo‐controlled clinical study,^48^ and a growing body of evidence points to its efficiency in the clinical management of acne,^49^, ^50^, ^51^ as well as in other pathological skin conditions.^52^, ^53^ Although the mechanism of nicotinamide's anti‐acne actions is still only partially understood,^53^ it is well‐evidenced that it does not activate HCA_2_;^16^, ^17^ therefore, it definitely does not share the above described lipostatic signalling pathway of NA. However, considering that NA can be metabolized to nicotinamide in vivo,^54^ it can be postulated that clinical efficiency of topically administered NA might even be superior to the one of nicotinamide, since theoretically, it could include both HCA_2_‐dependent direct, and, following its conversion to nicotinamide, HCA_2_‐independent, indirect anti‐acne effects.

As mentioned above, despite of its lipostatic and anti‐proliferative efficiency, NA was unable to prevent LPS‐induced pro‐inflammatory response of the sebocytes (Figure 2). It is noteworthy that, in light of our previous data obtained by using the non‐psychotropic phytocannabinoid (‐)‐cannabidiol, the observed anti‐inflammatory inefficiency of NA is not completely unexpected. Indeed, (‐)‐cannabidiol was found to exert lipostatic and anti‐proliferative actions through the activation of TRPV4 ion channels and the subsequent elevation of the [Ca^2+^]_IC_. Its anti‐inflammatory actions, however, were independent of the impact on the calcium homeostasis, and were mediated by an adenosine A_2A_ receptor‐dependent manner.^26^ Moreover, we have recently demonstrated that activation of another Ca^2+^‐permeable ion channel (namely TRPV3) suppressed SLG, and enhanced expression and release of several pro‐inflammatory cytokines.^42^ Thus, it is not surprising that NA was efficient in normalizing the SLG via the HCA_2_‐dependent elevation of the [Ca^2+^]_IC_, but failed to prevent the pro‐inflammatory action of LPS. Importantly, however, NA can still be effective in alleviating the acne‐accompanying pathological inflammation.^55^, ^56^, ^57^, ^58^

Indeed, as discussed above, NA appears to have negligible effects on the immune behaviour of human sebocytes (Figure 2), but through direct actions on professional immune cells, eg macrophages,^59^, ^60^ it still has the potential to exert beneficial effects in acne. Moreover, sebocytes were shown to regulate skin immune processes not only via direct cytokine, chemokine and adipokine release,^2^, ^3^, ^40^, ^55^, ^56^, ^58^ but also by the composition of the produced sebum.^4^, ^57^ Thus, although the exact impact of NA on the qualitative lipidome is to be dissected in further targeted studies, our data on the potent lipostatic effects of NA support the concept that it might beneficially influence cutaneous immune homeostasis when normalizing SLG.

One key remaining question is how exactly HCA_2_ activation can lead to Ca^2+^‐signals in human sebocytes. Although HCA_2_ is a G_i_‐coupled receptor, it is not unprecedented that its activation is followed by Ca^2+^‐influx.^10^, ^11^ Identification of the so far unknown Ca^2+^ permeable pore(s) promises to be of great clinical relevance, since it/they could also be targeted in the future anti‐seborrhea/anti‐acne drug development, especially, since unlike eg TRPV3,^42^ its/their activation results in pure sebostatic effects without triggering up‐regulation and release of pro‐inflammatory cytokines (Figure 6). Taken together, NA, most likely together with other HCA_2_ receptor agonists, might exhibit great translational potential in the management of acne and seborrhea. Placebo‐controlled clinical trials by using appropriate topical formulations delivering NA directly to the sebaceous glands and thereby minimizing unwanted side effects are therefore urgently invited to scrutinize if dermatological use of NA (and other HCA_2_ agonists) as topically administered agent provides valuable clinical benefit.

## CONFLICT OF INTEREST

CCZ owns an international patent on the SZ95 sebaceous gland cell line (WO2000046353).

## Data Availability

The data that support the findings of this study are available from the corresponding author upon reasonable request.
