# Supplementary material for: Nicotinic acid suppresses sebaceous lipogenesis of human sebocytes via activating hydroxycarboxylic acid receptor 2 (HCA2)
Source: J Cell Mol Med. 2019 Jul 5;23(9):6203–14. doi: 10.1111/jcmm.14505 (PMC6714165; doi:10.1111/jcmm.14505)
Supplement: Supplementary file 1 [file JCMM-23-6203-s001.pdf]

SUPPLEMENTARY DATA

Nicotinic acid suppresses sebaceous lipogenesis of human sebocytes via activating hydroxycarboxylic acid receptor 2 (HCA_2)

Arnold Markovics[1], Kinga Fanni Tóth[1], Katalin Eszter Sós[1,2], József Magi[1], Adrienn Gyöngyösi[3], Zoltán Benyó[4], Christos C. Zouboulis[5], Tamás Bíró[6,7,#,*], Attila Oláh[1#]

[#]These authors contributed equally.

[1]Department of Physiology, Faculty of Medicine, University of Debrecen, Debrecen, Hungary; [2]Laboratory of Cerebral Cortex Research, Institute of Experimental Medicine Hungarian Academy of Sciences, Budapest, Hungary; [3]Department of Immunology, Faculty of Medicine, University of Debrecen, Debrecen, Hungary; [4]Institute of Clinical Experimental Research, Semmelweis University, Budapest, Hungary; [5]Departments of Dermatology, Venereology, Allergology and Immunology, Dessau Medical Center, Brandenburg Medical School Theodor Fontane, Dessau, Germany; [6]DE-MTA "Lendület" Cellular Physiology Research Group, Department of Immunology, Faculty of Medicine, University of Debrecen, Debrecen, Hungary; [7]HCEMM Ltd., Szeged, Hungary

[*]CORRESPONDING AUTHOR:

Tamás Bíró, MD, PhD, DSc; DE-MTA "Lendület" Cellular Physiology Research Group, Department of Immunology, Faculty of Medicine, University of Debrecen; Egyetem square 1. Debrecen, H-4032, Hungary; e-mail: biro.tamas@med.unideb.hu; phone/FAX: +3652-417-159.

SUPPLEMENTARY FIGURES

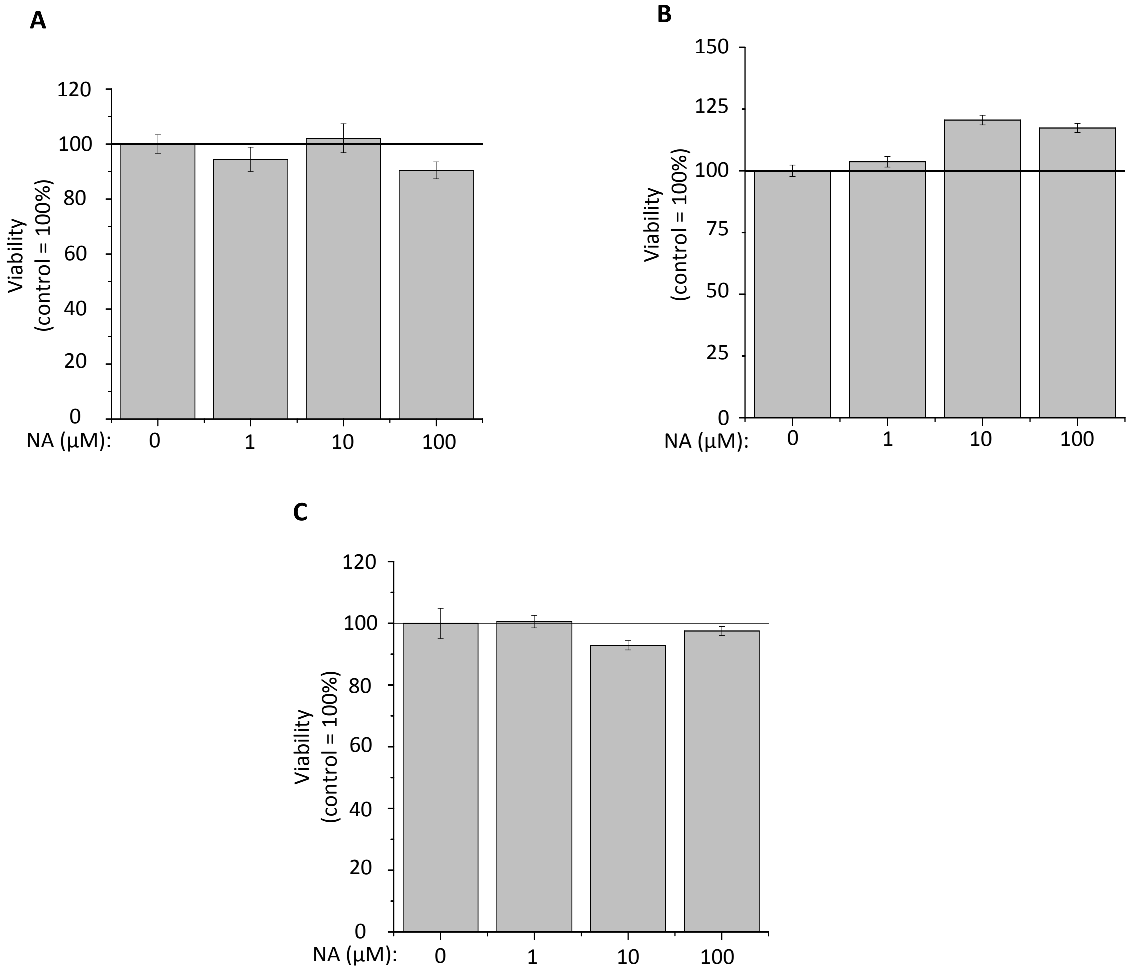

Supplementary Figure S1 *Up to 100 µM, NA does not influence viability of human sebocytes*

MTT-assays. Viability of SZ95 sebocytes was monitored following 24- (**A**), 48- (**B**), and 72-hr (**C**) treatments. Results are expressed in the percentage of the vehicle control (100%, solid line) as mean±SEM of four independent determinations. Two additional experiments yielded similar results.

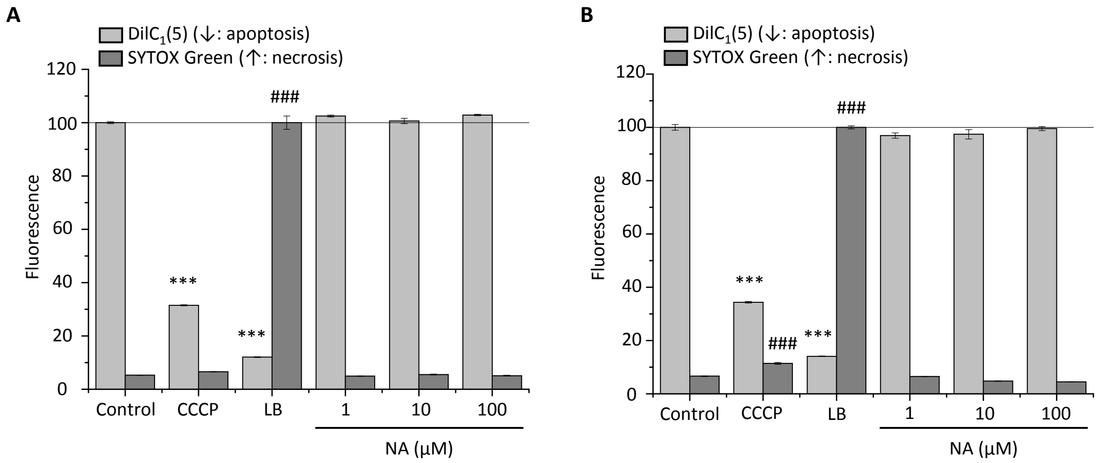

Supplementary Figure S2 *Up to 100 μM, NA can be applied without the risk of cytotoxicity*

Combined fluorescent DilC$_1$(5)-SYTOX Green labeling. To monitor apoptotic and necrotic cell death, SZ95 sebocytes were treated as indicated for 24 (**A**) or 48 (**B**) hours. Results are expressed in the percentage of the vehicle control (100%, solid line; DilC$_1$(5) apoptosis data) or in the percentage of the positive control (100%, solid line; SYTOX Green necrosis data) as mean±SEM of four independent determinations. Two additional experiments yielded similar results. *** and ### mark significant (*P*<0.001 in both cases) differences compared to the vehicle control group. **CCCP:** carbonyl cyanide m-chlorophenyl hydrazone (1:200; apoptosis positive control); **LB:** lysis buffer (1:100; positive control for necrosis); **NA:** nicotinic acid.

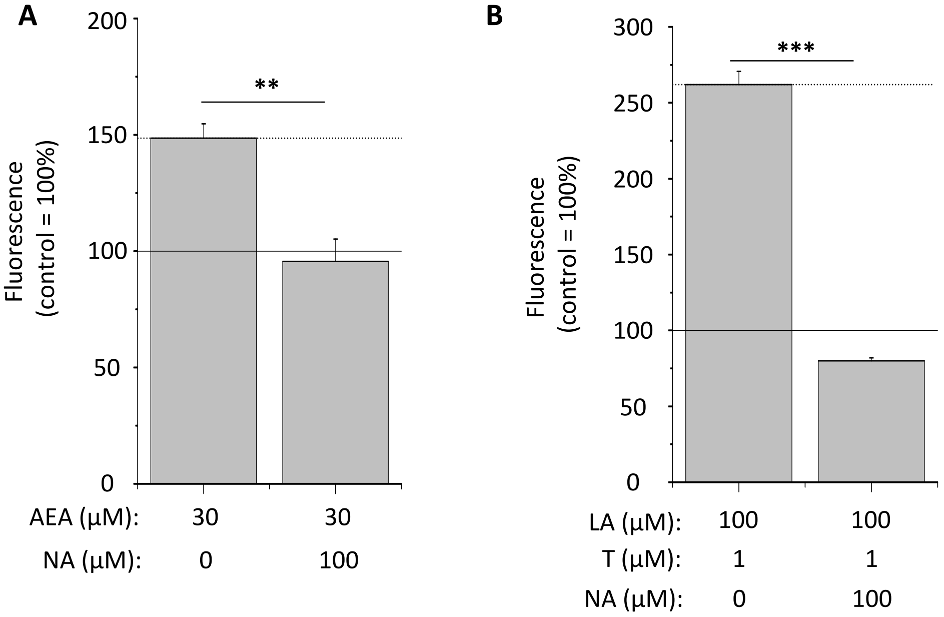

Supplementary Figure S3 *NA exerts universal lipostatic effects*

Nile Red assay. Lipostatic efficiency of NA was assessed following 48-hr treatments in the presence of AEA (**A**) or LA+T combination (**B**). Results are expressed in the percentage of the vehicle control (100%, solid line) as mean±SEM of four independent determinations. One additional experiment yielded similar results. ** and *** mark significant (*P*<0.01 and 0.001, respectively) differences, as indicated. **AEA:** anandamide; **LA:** linoleic acid; **NA:** nicotinic acid; **T:** testosterone.

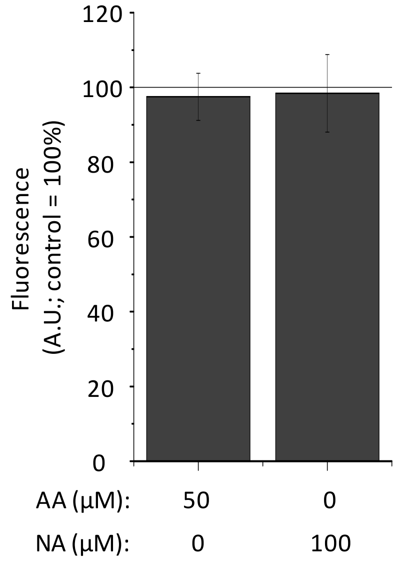

Supplementary Figure S4 *Neither AA, nor NA influence HCA_2 expression in human sebocytes*

Immunofluorescent labeling. HCA_2 expression was assessed following the indicated 24-hr treatments. Following appropriate background subtraction (for details, see the **Materials and methods** section) data of the green channel were expressed in the percentage of the vehicle control, and presented as mean±SEM of N=15-16 cells in each group. **A.U.:** arbitrary units.
